# Assessment of the psychometric properties of the Italian version of the perceptions of empowerment in midwifery practice scale-revised (PEMS-R-IT) in midwives

**DOI:** 10.18332/ejm/146587

**Published:** 2022-05-23

**Authors:** Simona Fumagalli, Sara E. Borrelli, Giovanni Galeoto, Francescaroberta Panuccio, Chiara Pignataro, Marianna Gottardi, Antonella Nespoli

**Affiliations:** 1 School of Medicine and Surgery, University of Milano-Bicocca, Monza, Italy; 2 Division of Midwifery, School of Health Sciences, University of Nottingham, Nottingham, United Kingdom; 3 Istituto di Ricovero e Cura a Carattere Scientifico, Neuromed - Istituto Neurologico Mediterraneo Pozzilli, Pozzilli, Italy; 4 Department of Human Neurosciences, Sapienza University of Rome, Rome, Italy; 5 San Daniele del Friuli Ospedale di Sant'Antonio, Azienda Sanitaria Universitaria Friuli Centrale, San Daniele, Italy

**Keywords:** midwifery, empowerment, psychometric properties, exploratory factor analysis, assessment tool

## Abstract

**INTRODUCTION:**

A higher degree of midwives’ empowerment is associated with greater job satisfaction and better midwifery care outcomes for women and their families. Empowered midwives are able to better empower women who in turn have a positive influence on the midwives’ empowerment. The aim of this study was the translation, cultural adaptation, and validation of the perceptions of empowerment in midwifery scale-revised (PEMS-R) in a group of Italian midwives.

**METHODS:**

The World Health Organization (WHO) method was adopted to achieve the PEMS-R Italian version. This process involved five steps: 1) forward translation, 2) expert panel translation, 3) back-translation, 4) pre-testing and cognitive interviewing, and 5) final version. The test’s internal consistency and validity were assessed by following international guidelines. Internal consistency was examined through Cronbach’s alpha (α) coefficient.

**RESULTS:**

The PEMS-R-IT was administered to 147 Italian midwives from northern Italy. Factor analysis of the 19 items, extracted 4 factors that explained 74.96% of the variance. The Student’s t-test for independent samples was used to identify a possible correlation between a higher/lower perception of empowerment and: 1) the education level, and 2) the years of experience of recruited midwives. No statistically significant differences were obtained in either case. The PEMS-R-IT was found to have a good internal consistency for each of its 4 subscales.

**CONCLUSIONS:**

The PEMS-R-IT is a valid and reliable tool, useful to assess midwives’ empowerment. It can be used in both clinical practice and research in order to investigate the level of empowerment of midwives within the Italian national context.

## INTRODUCTION

Empowerment is generally defined as the process of giving a person or group of people power and status in a particular situation^1^. Within healthcare organizational contexts, empowerment includes two different aspects: structural and psychological. The first is described as the ability to equip resources, provide access to information, support, learning and development^2^. The latter refers to the motivation and competence felt by an individual to actively fulfill work expectations^3^. The importance of empowerment within the midwifery profession has been highlighted internationally with the development of midwives’ sense of autonomy and subsequently their empowerment seen as a critical element to recruitment and retention, requiring attention and strengthening^4^. A higher degree of midwives’ empowerment is associated with better midwifery retention^5,6^, greater job satisfaction^7-10^, decreased burnout^3^, improvement of maternity care safety and outcomes^11-13^, and increased development of healthcare professionals’ full potential^14,15^. Empowered midwives are able to better empower women who in turn have a positive influence on the midwives’ empowerment^16,17^.

Within the Italian context, the Federation of Midwives reports 20500 midwives provide antenatal, intrapartum and postnatal care to women and families, with levels of autonomy varying at regional and national levels. To become a qualified midwife, an Undergraduate degree (180 university credits) is required. As part of postgraduate education, the Master’s degree (120 university credits) provides midwives with additional qualification within the education, leadership and/or research fields. The first level Master’s degree (60 university credits) is focused on more advanced skills. Most Italian midwives are employed via the public national health system and work predominantly within hospital maternity services and to a lesser extent in community. Midwifery-led units and independent midwives are not frequent options for women giving birth in Italy. Levels of empowerment of Italian midwives may be negatively impacted by continuing challenges with shortage and retention within the professional group^5,18^.

For the reasons highlighted above, it is important to investigate the perception of the level of empowerment of midwives. Matthews et al.^19^ developed the perceptions of empowerment in midwifery scale (PEMS), a 22-item scale for the evaluation of empowerment in midwives to asses autonomous practice, effective management and woman-centered practice. A revised version of the same tool (PEMS-R) was developed in 2015 by Pallant et al.^20^; further information about PEMS-R is included in the instrument section. The absence of a similar tool in Italy makes it difficult to assess the same characteristics of midwives’ empowerment in the Italian context. For this reason, the aim of this study was the translation, cultural adaptation, and validation of the PEMS-R in a group of Italian midwives. The Italian version of the scale would not only provide data about empowerment of midwives in Italy over time but also allow for cross-cultural comparison with other countries that have adopted the same tool^4,20-23^.

## METHODS

This work was carried out by a team of researchers from the University of Milano-Bicocca and from the Sapienza University of Rome, Italy. The research group has previous experience in scale validation^24,25^. After receiving consent from the developers of the original instrument, the scale was validated by undertaking the following steps, according to the World Health Organization (WHO) Translation Protocol^26^: 1) forward translation; 2) expert panel translation; 3) back-translation; 4) pre-testing and cognitive interviewing; and 5) final version.

### Instruments

The revised version of the empowerment in midwifery practice scale-revised (PEMS-R) was developed in 2015 by Pallant et al.^20^ including a pool of 19 items, divided into four, more easily interpretable subscales: autonomy/empowerment (4 items); manager support (5 items); professional recognition (5 items); and skills and resources (5 items). Total scores are calculated for each of the subscales by summing all the scores from each item, and then dividing by the number of items in the same subscale. Scores range from 1 (low level of empowerment), to 5 (high level of empowerment).

### Translation and cultural adaptation

The cultural adaptation process is vital when an instrument is used in different languages, contexts and times to reduce the risk of research bias^27^. The cultural adaptation process aims at achieving different language versions of a tool that are ‘conceptually equivalent in each of the target countries/cultures’, with a focus on conceptual meaning rather than simply on linguistic equivalence. A recognized method to achieve this is to use forward and back-translations^26^. Once the consent of the authors of the original article was received, the PEMS was translated from English to Italian following the ‘Translation and cultural adaptation of patient reported outcomes measures - principles of good practice’ guidelines^28^. The original English version was translated into three independent Italian translations by three English speaking health professionals. The results were then synthesized by an independent native speaker who had not been involved in the forward translations. Similarly, without having seen the original version of the tool, a group of three Italian translators then translated the questionnaire back into English. The original version and the back-translated version of the PEMS were then compared, and a panel of five Italian and expert midwives had to adapt the literally translated version of the tool to the Italian culture. For this reason, the final version has been corrected and modified in order to resolve any remaining spelling, diacritical, grammatical, or other errors, and to make the interpretation of the scores and the final statistics easier.

### Participants

Qualified midwives practicing in Italy were recruited as part of the pre-testing and cognitive interviewing phase. Midwives were recruited via three Italian Colleges of Midwives responsible for professional registration. An informative email was forwarded to potential participants, including a participant information sheet and a link to access the informed consent, scale and demographic questions. A member of the research team was available to answer any queries, provide more detailed information on the study and discuss potential participation.

### Ethics statements

The study was undertaken in accordance with ethical standards from the 1964 Declaration of Helsinki^29^ and its later amendments. The present study was exempt from IRB approval as per Institutional policy on validation studies.

### Statistical analysis

All the statistical analyses were carried out with the Statistical Package of Social Sciences (SPSS) 18.0. Reliability and validity of the Italian culturally adapted PEMS was assessed following the consensus-based standards for the selection of health status measurement instruments (COSMIN) checklist^30^.

Exploratory factor analysis is a multivariate analysis method that is applied to analyze the correlations between variables, in order to identify their latent structure (latent variables, not directly observable, which represent the common part of several observed variables). In this case, the exploratory factorial analysis was performed in order to establish whether the items of the PEMS-R-IT grouped together might obtain the same subscales as the original version.

The Student’s t-test for independent samples was used to verify whether the mean value of a distribution differs from a certain reference value. It is usually used in small samples, with a normally distributed population, when the standard deviation (SD) is unknown. In this study, the Student’s t-test was used to verify if there were differences in the perception of empowerment, in each of the subscales, depending on the midwives’ education background (undergraduate degree, Master’s degree, first level Master’s degree) and/or years of experience.

The internal consistency was examined using Cronbach’s alpha (α) in order to assess the interrelatedness of the items and the homogeneity of the scale. Cronbach’s α values higher than 0.70 were considered acceptable as an indicator of the homogeneity of the items within the total scale, establishing the reliability of the instrument.

## RESULTS

Following translation and cultural adaptation of the tool using the WHO translation Protocol^26^ the PEMS-R-IT was administered to 147 midwives recruited from hospitals, clinics or other services located mainly in Northern Italy. All results concerning the study sample are shown in Table 1.

**Table 1:** Characteristics of participants in the validity and reliability study of the Italian version of the perceptions of empowerment in midwifery scale-revised (PEMS-R-IT) in Italian midwives (N=147)

*Characteristics*	*Categories*	*n (%)*
**Education level**	Undergraduate degree	103 (70.1)
	Master’s degree	23 (15.6)
	First level Master’s degree	21 (14.3)
**Years of experience**	<5	61 (41.5)
	5–10	18 (12.2)
	>10	68 (46.3)
**Workplace**	Hospital	137 (93.2)
	Community	10 (6.8)

Exploratory factor analysis was used to estimate the construct validity of the 19-items PEMS-R-IT, extracting 4 factors that explained 74.9% of the variance. This result reflects the exploratory factorial analysis of the original article of the revised version, identifying the same 4 subscales. Exploratory factor analysis of the PEMS-R-IT is reported in Table 2.

**Table 2:** Factor analysis of the extracted items of the Italian version of the perceptions of empowerment in midwifery scale-revised (PEMS-R-IT) in Italian midwives

*Items*	*Autonomy/empowerment*	*Manager support*	*Professional recognition*	*Skills and resources*
I am an advocate for birthing women			0.784	
I empower birthing women through my practice			0.807	
I am involved in midwife-led practice			0.762	
I have autonomy in my practice			0.538	
I do not have a supportive manager	-0.876			
I am valued by the manager	0.912			
I have the back-up of the manager	0.930			
I am not recognized for my contribution to the care of birthing women by my manager	-0.853			
I have effective communication with management	0.841			
I am recognized as a professional by the medical profession		0.819		
I am recognized for my contribution to the care of birthing women by the medical profession		0.754		
I am not listened to by members of the multidisciplinary team		-0.779		
I have control over my practice		0.511		
I have support from my colleagues		0.673		
I am adequately educated to perform my role				0.846
I do not have the skills required to carry out my role				-0.871
I do not know what is my scope of practice				-0.771
I do not have adequate access to resources for staff education and training				-0.851
I have adequate access to resources for birthing women in my care				0.780
% Variance	38.4	14.6	12.1	9.8

**Figure 1:**
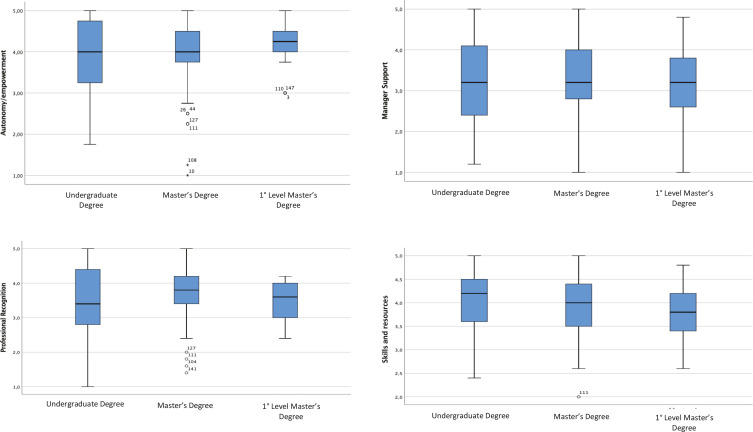
Stem and Leaf Plots representing median and variances of the score obtained by the 147 midwives divided by educational level in each subscale

**Figure 2:**
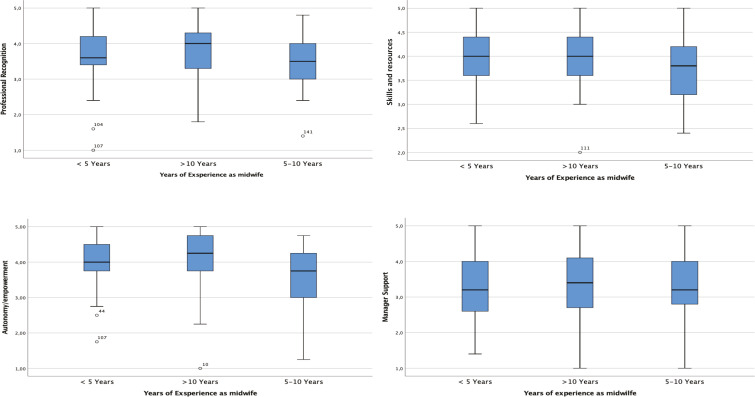
Stem and Leaf Plots representing median and variances of the score obtained by the 147 midwives divided by years of experience in each subscale

The Student’s t-test for independent samples identified the correlation between the midwives’ level of empowerment (higher/lower) and their education level and years of experience. Table 3 shows the mean score of the 147 midwives (with 95% CI) in each subscale, divided into education levels (undergraduate degree, Master’s degree, first level Master’s degree) and years of experience (<5, 5–10, and >10 years). The same results can graphically be observed in the stem and leaf plots, which allow to represent on the same graph five position measures: the minimum value, the first quartile, the median, the third quartile, and the maximum value of a variable. Figure 1 represents the distribution of PEMS-R-IT scores in the 4 subscales, based on the midwives’ education level. In the autonomy/empowerment subscale, the median line (which indicates the central value of the distribution) is 4.00 for the Master’s degree and the undergraduate degree, while it is slightly over 4.00 for the first level Master’s degree. The upper whisker extends upwards to the maximum value which is ≤1.5 times the interquartile range (IQR), while the lower whisker extends downwards to the smallest value ≥ 1.5 times the IQR. Potential outliers are represented with dots, concerning undergraduate degree and first level Master’s degree. Figure 2 represents the distribution of PEMS-R-IT scores in the 4 subscales, based on the midwives’ years of experience. In the autonomy/empowerment subscale, median values are slightly different according to the category investigated, with two outliers in the <5 years category and one outlier in the 5–10 years category.

**Table 3:** Mean scores of the midwives divided by education level and years of experience, in each subscale of the perceptions of empowerment in midwifery scale-revised (PEMS-R-IT) (N=147)

*Variable*	*Autonomy/empowerment*		*Manager support*		*Professional recognition*		*Skills and resources*	
	*Mean*	*95% CI*	*Mean*	*95% CI*	*Mean*	*95% CI*	*Mean*	*95 % CI*
**Education level**								
Undergraduate degree	4.04	3.88–4.19	3.31	3.12–3.50	3.75	3.60–3.90	3.91	3.80–4.01
Master’s degree	3.96	3.56–4.36	3.36	2.84–3.87	3.52	3.06–3.98	4.05	3.76–4.34
First level Master’s degree	4.15	3.87–4.43	3.13	2.62–3.64	3.49	3.21–3.78	3.74	3.45–4.02
**Years of experience**								
<5	4.07	3.91–4.23	3.24	3.00–3.48	3.65	3.46–3.85	3.91	3.76–4.07
5–10	3.66	3.22–4.11	3.14	2.56–3.73	3.43	3.01–3.84	3.77	3.45–4.10
>10	4.12	3.91–4.32	3.37	3.12–3.63	3.76	3.56–3.96	3.93	3.79–4.06

The Cronbach’s alpha of the PEMS-R-IT was analyzed for each of the four subscales, which were found to have good internal consistency. Autonomy/empowerment subscale was found to have an α value of 0.807, manager support 0.948, professional recognition 0.866, and skills and resources 0.767, showing the lowest value (Table 4).

**Table 4:** Cronbach’s alpha for each item of each subscale of the perceptions of empowerment in midwifery scale-revised (PEMS-R-IT) in Italian midwives

*Subscale*	*Item*	*Corrected item-total correlation*	*Cronbach’s alpha if item deleted*
**Autonomy/empowerment**	I am an advocate for birthing women	0.676	0.738
	I empower birthing women through my practice	0.652	0.750
	I am involved in midwife-led practice	0.659	0.747
	I have autonomy in my practice	0.545	0.797
Total subscale			0.807
**Manager support**	I do not have a supportive manager	0.850	0.937
	I am valued by the manager	0.893	0.929
	I have the back-up of the manager	0.931	0.922
	I am not recognized for my contribution to the care of birthing women by my manager	0.826	0.941
	I have effective communication with management	0.785	0.948
Total subscale			0.948
**Professional recognition**	I am recognized as a professional by the medical profession	0.794	0.810
	I am recognized for my contribution to the care of birthing women by the medical profession	0.805	0.807
	I am not listened to by members of the multidisciplinary team	0.699	0.835
	I have control over my practice	0.607	0.858
	I have support from my colleagues	0.542	0.871
Total subscale			0.866
**Skills and resources**	I am adequately educated to perform my role	0.631	0.694
	I do not have the skills required to carry out my role	0.663	0.691
	I do not know what my scope of practice is	0.457	0.759
	I do not have adequate access to resources for staff education and training	0.549	0.746
	I have adequate access to resources for birthing women in my care	0.516	0.733
Total subscale			0.767

## DISCUSSION

The PEMS-R is a specific assessment tool for the evaluation of empowerment in midwives. The aims of this study were the translation, cultural adaptation, and validation of the perceptions of empowerment in midwifery scale-revised (PEMS-R-IT) in a group of Italian midwives. The translation of the original PEMS-R was performed applying internationally recognized methods^17,18^.

A factor analysis was performed to identify whether the items of the PEMS-R-IT could be grouped under the same 4 subscales (autonomy/empowerment, manager support, professional recognition, and skills and resources) developed by Pallant et al.^20^ as part of the PEMS-R validation. Our factor analysis resulted to be in agreement with the one performed by Pallant et al.^20^ in the New Zealand setting.

Lukasse and Pajalic^21^ reported that empowerment levels (especially within the factors supportive management and autonomous professional role) are significantly lower for midwives working in a hospital setting compared to those not working in a hospital setting. This can be explained by the work of Hildingsson et al.^4^, suggesting that midwives’ sense of empowerment increases when they are supported to be autonomous practitioners. However, our results cannot confirm this, as a stratified analysis by workplace could not be performed due to non-comparable sample sizes between the two groups, with 137 (93.2%) hospital midwives and only 10 (6.8%) community midwives.

The Student’s t-test for independent samples was used to identify a possible correlation between empowerment and education level, and between empowerment and years of experience. In the first case, as shown in Table 3, no statistically significant values were shown between the mean scores obtained in the 3 categories of education level taken into consideration (undergraduate degree, Master’s degree, first level Master’s degree). The higher/lower level of education seemed to not significantly affect the higher/lower perception of empowerment from the statistical analysis we performed. However, higher differences in the level of empowerment means between education levels was noted for autonomy/empowerment, as shown in Figure 1. Lukasse and Pajalic^21^ observed that postgraduate education resulted in a significantly higher score for an autonomous professional role but not for experiencing supportive management or feeling equipped for practice. Matthews et al.^19^ and Pallant et al.^20^ describe that midwifery empowerment is affected by midwives’ perception of their knowledge, competence, skills and ability to access the required resources to fully work across their scope of practice and provide quality woman centered care.

The same non-statistically significant result can be observed by analyzing the mean scores obtained by years of experience. As shown in Table 3, there are no statistically significant differences in the 4 subscales for the 3 considered categories (<5, 5–10, and >10 years), thus deducing that the years of work experience do not seem to positively/negatively influence the perception of empowerment of midwives. The same results and the same considerations are obtained by analyzing the stem and leaf plots, in which medians and variance are analyzed. In all the analyzed graphs, the median values are mostly equal or present some differences that cannot be considered statistically significant. This can be explained by the fact that in the Italian midwifery care system there are no grades or levels based on years of work or experience, as also reported by Hildingsson et al.^4^ within the Swedish context. Lukasse and Pajalic^21^ found that midwives with more than 20 years’ experience had considerably higher scores for all subscales.

The results of our study suggest that the Italian version of the PEMS-R (PEMS-R-IT) is a valid and reliable tool for assessing empowerment in Italian midwives, confirming the results obtained by Pallant et al.^20^, which suggested an alternative four subscale structure for the PEMS to the one suggested by the original developers of the tool^19^. As well as the original version, the PEMS-R-IT showed good internal consistency in all four subscales, with the highest alpha value in the manager support subscale (α=0.948), and the lowest value in the skills and resources subscale (α=0.767). Moreover, in the corrected item-total correlation, item 17 (‘I have control over my practice’) appears to be the least correlated within the entire scale, with α=0.457.

The work undertaken by Hildingsson et al.^4^ comparing midwifery empowerment in three countries (Australia, New Zealand, and Sweden) acknowledges the importance of considering midwifery care culture and related factors within different contexts and healthcare systems, supporting the value of our PEMS-R-IT validation.

### Limitations

This study has some limitations that need to be considered and improved in subsequent studies. There is no correlation with a gold standard because no other tools relating to the measurement of empowerment in midwives have been identified. The scores of the PEMS-R-IT did not find any statistically significant difference of empowerment levels based on years of experience and education level; this could be due to the small sample size and non-comparable groups. A self-reporting questionnaire exposed the data to the potential of social desirability effects in regard to midwives’ perception of own practice.

## CONCLUSIONS

The PEMS-R-IT has proven to be a valid, reliable, and rapid to administer tool useful for investigating and measuring the level of empowerment perceived by Italian midwives in their workplace. It is a new tool useful in both clinical practice and research to underline the importance of promoting the empowerment of midwives and promote the development of self-determination and professional fulfilment. Future studies using the PEMS-R-IT may test the tentative relationships suggested by this study using larger appropriate sub-groups (e.g. level of education, years of experience, and workplace) and larger samples from each geographical region. This may help in identifying any differences in the perception of empowerment due to the variance in socioeconomic and geographical realities. Future validations could include a test-retest analysis to the statistical processing of the data, in order to deepen and make the PEMS-R-IT more reliable. Further research should also focus on the factors influencing levels of empowerment.

## Data Availability

The data supporting this research are available from the authors on reasonable request.
